# Lethal End of Spectrum of Clots-Thrombotic Storm

**DOI:** 10.1155/2018/7273420

**Published:** 2018-05-27

**Authors:** Muhammad Asim Rana, Ahmed F. Mady, Abdullah Ali Lashari, Rehab Eltreafi, Nicola Fischer-Orr, Kamal Naser

**Affiliations:** ^1^Critical Care Department, Bahria Town International Hospital, Lahore, Pakistan; ^2^Critical Care Department, King Saud Medical City, Riyadh, Saudi Arabia; ^3^Acute Medicine Department, King's Mill Hospital, Sutton in Ashfield, UK

## Abstract

Thrombotic storm (TS) is a rare, acute, hypercoagulable state characterized by multiple thromboembolic events affecting at least two different areas of the vascular system/organs over a short period of time. Typical triggers include inflammation, infections, minor trauma, surgery, pregnancy, and the puerperium. A single thrombotic event can set off a number of thromboembolic events, often including unusual locations like hepatic, portal, or renal veins, skin* (purpura fulminans)*, adrenal glands, and cerebral sinus venous thrombosis. Usually, younger female patients are affected; in some patients, there is an association with an autoimmune disorder like lupus erythematosus, and they show evidence of antiphospholipid antibodies or other phenotypic expressions of anticoagulation disorders. The majority of patients have no previous history of thromboembolism. As the diagnosis of thrombotic storm relies solely on clinical symptoms with a lack of specific diagnostic tests, this can result in a delay of diagnosis. The treatment consists of uninterrupted lifelong anticoagulation. Sometimes immunomodulatory therapies have been used. The distinction between extensive thrombotic events like Heparin Induced Thrombosis (HIT), Thrombotic Thrombocytopenic Purpura (TTP), Antiphospholipids Syndrome (APS), and TS can sometimes be difficult, and the etiology of TS remains uncertain.

## 1. Case Report

A 27-year-old Ethiopian female was brought to the Emergency Department (ED) with a short history of rapidly increasing confusion, deteriorating consciousness, and difficulty in breathing. Her Glasgow Coma Scale (GCS) in ED was 8/15, temperature 37.5°C, heart rate 138 bpm, regular blood pressure 95/79 mmHg, and respiratory rate 38 per minute with oxygen saturation 88% on 15 L/min oxygen via non-rebreathing mask. The examination of the chest, cardiovascular system, and abdomen was unremarkable. Because of a rapidly worsening GCS and increasing respiratory failure, she was electively intubated and ventilated in ED.

## 2. Initial Lab Results in ED

Initial laboratory results in ED showed a severe metabolic acidosis pH of 7.0, lactate 10 mmol/L, leukocytosis 28.9 × 10^9^/L with a left shift, raised renal functions (creatinine 227 mmol/L, urea 22 mmol/L), and abnormal liver function tests (ALT 386 U/L, AST 802 U/L, total bilirubin 6.7 *μ*mol/L, raised CK 579 U/L, CK-MB 57 U/L, Trop-I 3.30 ng/ml, and LDH 5884 U/L). The coagulation profile was also normal (PT 11.5, INR 0.8, APTT 35). The urine analysis was positive for blood, proteins, nitrites, and bacteria. Her initial chest X-ray was showing some bilateral infiltrates.

## 3. ICU Course and Workup

She was transferred to the ICU with an initial diagnosis of septic shock (hypotension, altered sensorium, and tachypnea) with a urinary tract infection as a source. In the ICU, the shock parameters worsened; her inotropic requirement was increasing as well as ventilatory and oxygen requirements.

Her lab results including renal and liver functions and hypoxia continued to deteriorate despite adequate hydration and antibiotics. Initially, piperacillin/tazobactam (loading dose of 4.5 g and maintenance dose of 2.25 g 8-hourly) and linezolid (600 mg twice a day intravenously) were started. Antibiotics were later upgraded to imipenem/cilastatin 500 mg 6-hourly and linezolid was continued. Her HIV and hepatitis serology was negative. The hemolytic panel including the reticulocyte count, haptoglobins, and peripheral smear was normal except for the raised LDH which gradually showed a decreasing trend. Her D-dimers and fibrinogen were elevated at 1800 ng/ml and 600 mg/dl, respectively.

The bedside echo cardiogram showed dilated right ventricular and tricuspid regurgitation indicating acute pulmonary hypertension. She was started on intravenous heparin therapeutic dose, for the treatment of suspected pulmonary embolism. An unprovoked pulmonary embolism (PE) in a young female led us to think of vasculitis and thrombophilia as possible causes. Although her personal and family history were negative for thrombophilia and connective tissue disorders, an extensive workup including ANA (less than 1.0 IU, negative), anti-dsDNA (less than 10 IU/ml, negative), complement factors C3 (0.689 grams/L) (Ref. 0.9–1.8) and C4 0.219 grams/L (Ref. 0.1–0.4), c-ANCA, p-ANCA, antiphospholipids including anticardiolipin antibodies, IgG less than 8.0 GP (Ref. 0.0–15.0 GPL Units) and IgM 5.4 MPL (Ref. 0.0–7.0 M Phospholipid Units), *β*2-glycoprotein IgA less than 3.1 (Ref. 0.0–3.5 A phospholipid units), IgG 7.8 (Ref. 0.0–10.0 G Phospholipid Units), IgM 8.1 (Ref. 0.0–12.0 M Phospholipids Units), lupus anticoagulant 22 GPL units (Ref. 20–39 G phospholipid units), Factor V Leiden 72% (Ref. 75–150% of normal), protein C 100 (65–135 IU/dL), and S greater than 63 U/dL and antithrombin III 105% (Ref. 80–120). So, all the results discussed above were within normal limits.

**Figure 1:**
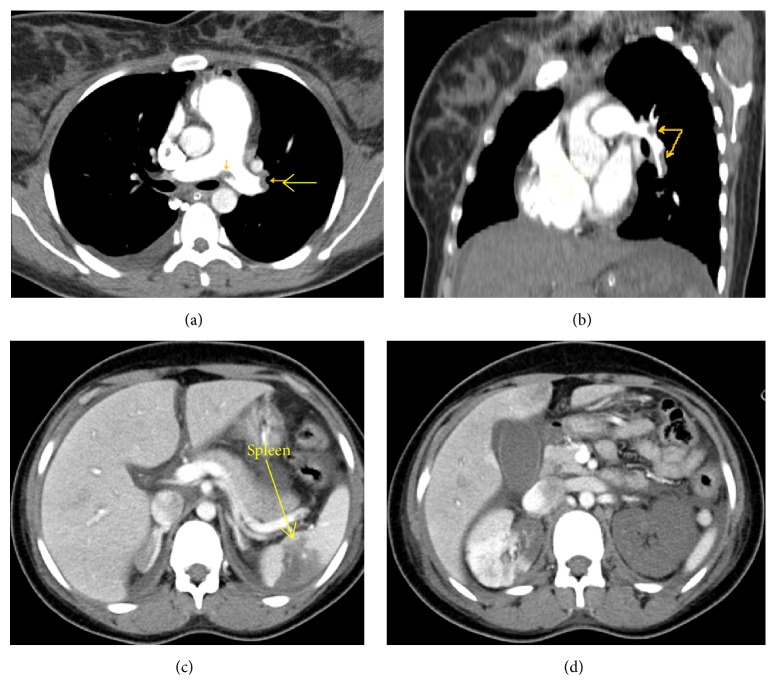
CECT PE Protocol showing bilateral massive pulmonary embolism (yellow arrows). CECT abdomen showing infarction in spleen (c). In (d), partly infarcted right and totally infarcted kidneys are also seen with partial opacification of inferior vena cava (d).

**Figure 2:**
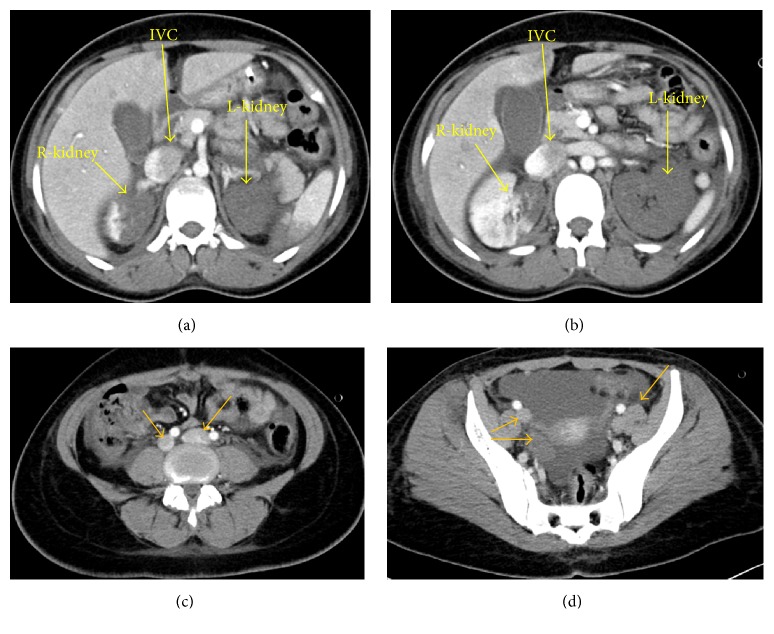
CECT abdomen showing partial infarction of the right kidney, totally nonperfused hypodense (completely infarcted) left kidney with thrombosis of distal left renal artery and proximal left renal vein. Inferior vena cava is seen partially filled with contrast (a, b) while nonenhancement is evident in distal to left renal vein. Thrombosis of inferior vena cava is seen extending into bilateral internal and external iliac veins and then into bilateral femoral veins (yellow arrows) (c, d).

On the third day of her ICU admission, she showed some improvement in terms of a reduced requirement of inotropic support and a reduction in ventilator settings and her CT chest PE Protocol was done which showed extensive bilateral pulmonary embolism. The pulmonary trunk was dilated with evidence of a saddle-shaped central filling defect seen at the bifurcation of the right and left main pulmonary arteries extending to their lobar and segmental branches bilaterally (Figures 1(a), 1(b), 1(c), and 1(d)). There were patchy alveolar air space and ground glass opacities seen bilaterally suggestive of an infectious process with mild right pleural effusion.The CT abdomen and pelvis with contrast showed multiple areas of thrombosis including a left renal global infarction with a thrombosed left renal artery and vein (Figures 1(a), 1(b), 1(c), and 1(d)). There were right renal and splenic hypodense areas secondary to infarctions. Noncontrast opacification of the IVC as well as the iliac veins indicated arterial and venous thrombosis (Figures 2(a), 2(b), 2(c), and 2(d)).

**Figure 3:**
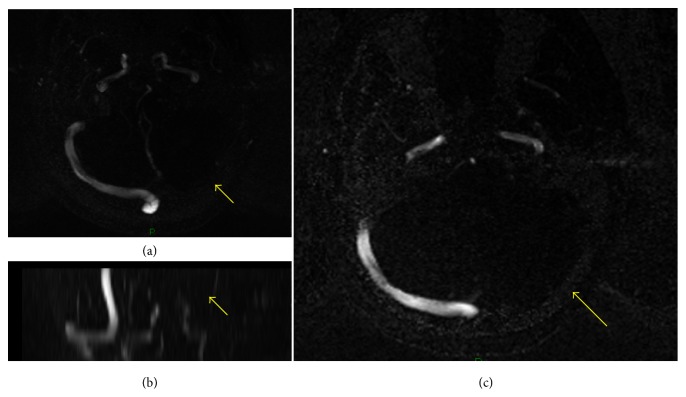
Loss of flow signal intensity in left lateral sinus which is showing heterogeneous appearance on FLAIR and T2-weighted images suggesting left lateral sinus thrombosis with venous infarction.

As her CT brain was showing brain edema, we carried out MR-A/V to rule out cerebral venous sinus thrombosis showing multiple cerebellar, brainstem, pons, and upper parietal bilateral cortical lesions with loss of flow signal intensity in the left lateral sinus showing heterogeneous appearance on FLAIR and T2-weighted images suggesting a left lateral sinus thrombosis with venous infarction (Figures 3(a), 3(b), and 3(c)).

## 4. Outcome

Taking her age and multiple arterial and venous thrombus formations in different vascular regions into consideration, a diagnosis of thrombotic storm was considered according to the criteria set by Kitchens et al. [1] (see Table 1). The patient was maintained on unfractioned heparin and other organ supports but unfortunately she died of therapy refractory multiple thrombi and overlying sepsis with septic shock leading to ARDS and multiorgan failure on the seventh day of her admission to the ICU.

## 5. Discussion

Thrombotic Storm (TS) was first described by Kitchens [2] as a rare, extreme, and often lethal clinical entity characterized by a series of thrombotic events which spread over a short span of time involving the arterial and venous circulatory beds in diverse and unusual sites [1, 2].

As widespread thrombosis is the key feature of TS, it brings this syndrome closer to other clinical entities known for hypercoagulable states and extensive thrombotic events like Antiphospholipid Syndrome (APS), Protein C and S Deficiency [3], Factor V Leiden, Heparin Induced Thrombocytopenia (HIT), Thrombocytopenic Purpura (TTP), and Hemolysis Elevated Liver enzymes and Low Platelets (HELLP) syndrome. The literature shows that antithrombin deficit [4] and endothelial damage are also involved [5]. In fact, TS has been diagnosed in association with the abovementioned disorders, which makes the diagnosis a conundrum as a clear link has yet to be established [3–14].

Some literature points towards some possible triggers like infection, trauma, surgery, and pregnancy, but so far no definite association could be pinpointed.

The trigger is postulated to develop certain chemical compounds which may come from different cellular origins, but they work synergistically to promote or create a prothrombotic environment in susceptible individuals [12–14].

It has been hypothesized by the thrombotic storm study group that these patients who go through these devastating thrombotic events might harbour some yet unknown risk factors which the study group aims to discover [1, 2].

TS is a rare condition; still, it is essential that it is recognized early due to its life-threatening nature and it is differentiated from the abovementioned conditions as the treatment strategy may be quite different; for instance, HIT demands a different anticoagulant to be used and TTP is treated with plasmapheresis [6–10].

Kitchen and his thrombotic storm study group have not defined this syndrome by any specific biochemical markers, tests, or any specific pattern of hypercoagulability but rather suggested that the syndrome should be recognized by its unique clinical course [1, 2, 12], so TS is, regardless of the cause, diagnosed at the bedside of these critically ill patients [1, 2].

The criteria proposed by the thrombotic storm study group (see Table 1) encircle the important features of young age, acute two or more arterial or venous thromboembolic locations, with or without microangiopathy, typically in a short span of time (days to 1-2 weeks), unusual location (other than pulmonary embolism, lower extremity deep vein thrombosis, myocardial infarction, and stroke) such as thrombosis of hepatic, cerebral, portal, or renal veins, skin, and adrenal glands. The criteria also stress upon the features like recurrence, refractory nature of the thrombotic cascade to acute therapy, exacerbation by inadequacy or interruption in treatment, and frequent presence of an initiating event like inflammation, trauma, surgery, pregnancy, and infection [1–3].

Our patient was diagnosed on the same criteria and this was preceded by a urinary tract infection.

Considering the core problem of thrombosis and taking evidence from the published literature favouring an aggressive anticoagulation therapy in Catastrophic Antiphospholipid Syndrome (CAPS), it has been suggested that the anticoagulation therapy is the essential treatment on which the survival of the patient depends [1, 3].

The anticoagulant treatment should be initiated immediately once TS is suspected as diagnosis at full therapeutic dosing using conventional monitoring goals and should be continued uninterrupted as interruption in anticoagulation has proven to exacerbate the thrombosis cascade. This is because fresh clots are known to contain and secrete thrombin and can aggravate the process of clot formation if anticoagulation is inadequate or interrupted [15].

Apart from anticoagulation, other interventions like thrombectomy and/or catheter-directed thrombolysis have been tried. In Piccin et al.'s review of 8 case studies, pharmacomechanical thrombolysis via an interventional radiologic approach, local catheter-directed and systemic tPA administration, surgical thrombectomy, or a combination of these modalities was used in different children [16].

The majority of the available literature advises against the use of an Inferior Vena Cava (IVC) filter in TS; also the available data is limited. The most important indication for the use of IVC filters in both adults and children is the prevention of PE in patients with lower limb VTE in whom systemic anticoagulation is contraindicated. Other relative indications are usually failure of anticoagulation therapy manifested by recurrent pulmonary embolism (PE), or propagation of deep vein thrombosis (DVT) and inability to achieve or maintain adequate anticoagulation [17, 18].

It should also be emphasized that there is only limited data available in current literature in the form of case reports, case series, and retrospective reviews that combine antithrombotic, pharmacomechanical thrombectomy, the use of Trellis Thrombectomy System (a novel treatment approach to clear thrombi from the ileofemoral vein), systemic thrombolysis, and immunomodulatory therapies in management of thrombotic storm [1–3, 16, 19, 20].

The long-term prognosis, once the thrombotic cascade stops and resolves, is generally very good. For patients who survive the thrombotic storm, lifelong anticoagulant therapy is recommended, either using a low molecular weight heparin (LMWH) or an oral vitamin K antagonist, since this is the only way to avoid recurrent thrombosis [21].

Our case highlights the need for a broad initial diagnostic approach once thrombotic storm is suspected. The clinician either in the admission areas (ED, Acute Medicine) or in intensive care should be alerted when dealing with younger patients, especially females presenting with episodes of thrombosis at unusual sites or recurrent thrombotic episodes in order to diagnose TS early and have a low threshold to start therapeutic anticoagulation usually with intravenous heparin. Similarly, it is needless to stress the fact that the anticoagulation should be continuous, uninterrupted, and within therapeutic range to stop the coagulation cascade and to prevent a devastating outcome.

Other conjunctive measures can be used depending upon the availability of modality and expertise in the center dealing with such cases.

## 6. Learning Pearls

- Thrombotic Storm is a rare, acute, hypercoagulable entity with rapidly spreading thrombotic events in unusual places with a preference for young females in conjunction with inflammation, trauma, surgery, infection, and pregnancy.
- The rarity of the disease and the lack of specific biochemical diagnostic tests will undoubtedly delay making the diagnosis. In this situation, the key to prompt diagnosis making and timely treatment is the physician's awareness of TS.
- Once suspected, the thrombotic storm study criteria should be used to help establish the diagnosis.
- Prompt and continuous anticoagulation remains the key of treatment which needs to be continued for life once the initial critical situation is over to avoid recurrences.

## Figures and Tables

**Table 1:** Diagnostic criteria of thrombotic storm reproduced with permission from Kitchens et al.

Thrombotic Storm Diagnostic Criteria
Typically encountered characteristics
Younger age *plus *2 or more of the following criteria:
Acute, 2 or more arterial or venous thromboemboli, with
or without thrombotic microangiopathy, ^*∗*^typically in a
compressed period of time (1-2 weeks) yet may recur
from time to time over years.
Unusual location^†^
Progressive/recent unexplained recurrence
Refractory to acute therapy or atypical response to
therapy
Exacerbated by inadequate or interrupted treatment (eg,
subtherapeutic anticoagulation)
Frequently preceded by an initiating event (“trigger”)^‡^
Characteristics usually not encountered
Cancer (excluding minor skin cancers)
Myocardial infarction in the setting of advanced coronary
artery disease
Cocaine use associated with symptom onset
Expected thrombotic complications associated with
intravascular devices
Known paroxysmal nocturnal hemoglobinuria or
myeloproliferative disorder
Multi-trauma/severe trauma (eg, multiple limb injury)
Premorbid clinical status before development of thrombotic
complications

^*∗*^Thrombotic microangiopathy defined as microvascular thrombosis (arteriole, venule, capillary) on tissue pathology. ^†^Unusual locations would include thromboembolic complications other than pulmonary embolism, lower extremity deep venous thrombosis, myocardial infarction, and stroke such as thrombosis of hepatic, cerebral, portal, or renal veins, skin (purpura fulminans), and adrenal glands. ^‡^Such as pregnancy, surgery, trauma, infections, and inflammatory states.

## Abbreviations

ED: Emergency Department
ICU: Intensive Care Unit
PE: Pulmonary Embolism
IVC: Inferior vena cava
IU: International Units.
